# Overdose Education and Naloxone Distribution Within Syringe Service Programs — United States, 2019

**DOI:** 10.15585/mmwr.mm6933a2

**Published:** 2020-08-21

**Authors:** Barrot H. Lambdin, Ricky N. Bluthenthal, Lynn D. Wenger, Eliza Wheeler, Bryan Garner, Paul Lakosky, Alex H. Kral

**Affiliations:** ^1^RTI International, Research Triangle Park, North Carolina; ^2^University of California San Francisco; ^3^University of Washington, Seattle, Washington; ^4^University of Southern California, Los Angeles, California; ^5^North America Syringe Exchange Network, Tacoma, Washington.

Syringe service programs (SSPs), which provide access to sterile syringes and other injection equipment and their safe disposal after use,* represent a highly successful human immunodeficiency virus (HIV) prevention intervention. SSPs are associated with a 58% reduction in the incidence of HIV infection among persons who inject drugs (*1*). In addition, SSPs have led efforts to prevent opioid overdose deaths by integrating evidence-based opioid overdose education and naloxone distribution (OEND) programs (*2*–*4*). OEND programs train laypersons to respond during overdose events and provide access to naloxone and directions for drug delivery (*2*–*4*). SSPs are ideal places for OEND because they provide culturally relevant services designed to reach persons at high risk for experiencing or observing an opioid overdose. A 2013 survey found that only 55% of SSPs in the United States had implemented OEND (*5*). To characterize current implementation of OEND among SSPs, and to describe the current reach (i.e., the ratio of persons who received naloxone per opioid overdose death and the ratio of naloxone doses distributed per opioid overdose death) of SSP-based OEND programs by U.S. Census division,^†^ a survey of known U.S. SSPs was conducted in 2019, which found that 94% of SSPs had implemented OEND. In addition, the reach of SSP-based OEND programs varied by U.S. Census division. Scaling up of SSP-based OEND delivery programs could be a critical component for areas of the country with high opioid overdose death rates and low reach.

The North America Syringe Exchange Network (NASEN)^§^ has provided technical and resource support to SSPs for the past 3 decades and as part of this effort maintains a database of all SSPs in the United States. In February 2019, all 342 SSPs in NASEN’s database were sent an e-mail asking organizational directors or their designee to participate in an online survey. If an SSP did not respond, additional e-mail or telephone follow-up was conducted to encourage participation. SSPs completing the online survey received a $50 honorarium. Opioid overdose deaths were identified using the *International Classification of Diseases, Tenth Revision* codes X40–X44 (unintentional overdose death); X60–X64 (intentional self-harm); X85 (assault [homicide]); or Y10–Y14 (undetermined intent), where the multiple cause of death codes included T40.0 (poisoning by opium), T40.1 (poisoning by heroin), T40.2 (poisoning by other opioids), T40.3 (poisoning by methadone), T40.4 (poisoning by other synthetic narcotics), or T40.6 (poisoning by other and unspecified narcotics). Opioid overdose deaths and opioid overdose death rates from 2017 were aggregated for the nine U.S. Census divisions, using publicly available data on population and opioid overdose deaths from CDC’s National Center for Health Statistics (*6*). SSPs were asked how many persons received naloxone and how many naloxone doses were distributed from their program in the past 12 months. The reach of SSP-based OEND programs for the nine U.S. Census divisions was estimated using two calculations: 1) the number of persons provided naloxone in the previous 12 months divided by the number of opioid overdose deaths in 2017 and 2) the number of naloxone doses distributed during the previous 12 months divided by the number of opioid overdose deaths in 2017. For both calculations, a higher ratio indicates greater reach. These two metrics were used to approximate the extent to which SSP-based naloxone distribution met the underlying need as determined by the number of opioid overdose deaths in the preceding calendar year. Data were analyzed using Stata (version 15.1; StataCorp). All study procedures were reviewed and approved by a federally accredited Institutional Review Board at RTI International.

Among the 342 known SSPs operating at the beginning of 2019, 263 (77%) responded to the online survey; of these, 247 (94%) had an OEND program, 160 (65%) of which had been implemented since 2016 (Figure 1). With regard to phases of OEND implementation, 173 (66%) responding SSPs had been implementing OEND for 12 months or more, 74 (28%) had implemented OEND within the last 12 months, eight (3%) were actively preparing for OEND implementation, and eight (3%) were exploring OEND implementation (Table). Of the 16 SSPs not yet offering OEND, four had previously implemented naloxone distribution but stopped because of an inadequate naloxone supply or funding.

**FIGURE 1:**
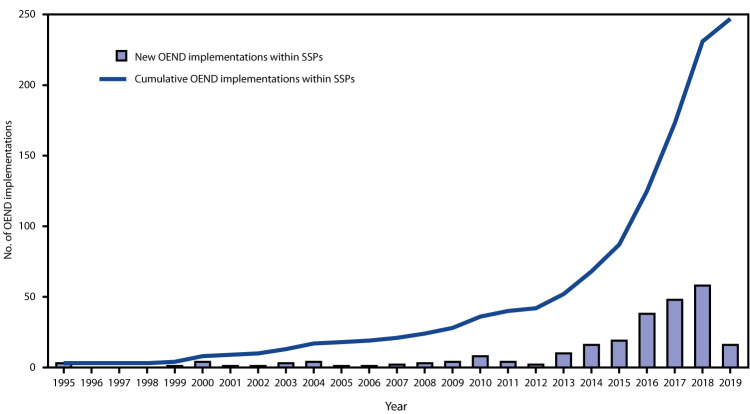
Number of new and cumulative overdose education and naloxone distribution (OEND) implementations within syringe service programs (SSPs),*^,†^ by year — United States, 1995–2019 * Data displayed are derived using responses from 263 of 342 SSPs throughout the United States. ^†^ Participating SSPs were identified by using the North America Syringe Exchange Network database.

**TABLE:** Characteristics of syringe service program (SSP) respondents (N = 263)* — United States, 2019

Characteristic	No. (%)
**U.S. Census division^†,^** ^§^	
East North Central	40 (15)
East South Central	13 (5)
Middle Atlantic	10 (4)
Mountain	28 (11)
New England	24 (9)
Pacific	83 (32)
South Atlantic	44 (16)
West North Central	15 (6)
West South Central	6 (2)
**Provide overdose prevention education^†^**	258 (98)
**Stage of OEND implementation^†^**	
Exploration	8 (3)
Preparation	8 (3)
Early implementation (<12 months)	74 (28)
Sustained implementation (≥12 months)	173 (66)
**Receive health department funding for OEND^¶^**	142 (57)
Local community support** for OEND^¶^, median (IQR)	80 (70–90)
**Naloxone offered every time syringe services offered^¶^**	191 (77)
**Number of days offering OEND in past 28 days,^¶^ median (IQR)**	15 (6–20)
**Naloxone refills provided as often as participants ask^¶^**	214 (87)
**Proactive refill system^¶^**	199 (80)
**Ran out of naloxone in past 3 months^¶^**	45 (18)
**Rationed naloxone in the past 3 months^¶^**	61 (25)
**Data system for OEND** ^¶^	
No data collected	9 (4)
Data collected via paper forms, then stored	50 (20)
Data collected via paper forms, then entered into database	149 (60)
Electronic data entry	29 (12)
**No. of programs by count of persons provided naloxone in the past 12 mos** ^††^	
Small (<100)	63 (27)
Medium (100–499)	76 (32)
Large (500–999)	36 (15)
Very large (≥1000)	62 (26)
**No. of programs by count of naloxone doses distributed in the past 12 mos** ^††^	
Small (<250)	76 (32)
Medium (250–999)	48 (20)
Large (1,000–9,999)	99 (42)
Very large (≥10,000)	14 (6)

**Abbreviations:** IQR = interquartile range; OEND = overdose education and naloxone distribution.

* Participating SSPs were identified by using the North America Syringe Exchange Network database.

^†^ Of the 263 SSPs that responded to the survey.

^§^ Region classification was determined by using the U.S. Census Bureau’s Census Regions and Divisions of the United States. https://www2.census.gov/geo/pdfs/maps-data/maps/reference/us_regdiv.pdf.

^¶^ Of the 247 SSPs that had implemented OEND.

** Respondents were asked to characterize the local community support for OEND on a scale of 1–100.

^††^ Of the 237 SSPs that had implemented OEND and reported naloxone distribution data.

Among the 247 SSPs with an OEND program, 191 (77%) offered OEND every time syringe services were offered, and 214 (87%) provided naloxone refills as often as participants requested them (Table). SSPs reported offering OEND for a median of 15 of the past 28 days. Only 29 (12%) SSPs entered OEND data directly into an electronic data system. During the preceding 12 months, 237 (96%) of 247 SSPs with OEND programs reported distributing 702,232 naloxone doses, including refills, to 230,506 persons (an average of 3 doses per person). Sixty-two (26%) SSPs reported distributing naloxone to >1,000 persons in the last 12 months; these programs had distributed naloxone to 186,603 laypersons, who represented 81% of all recipients in the past 12 months. Overall, 14 (6%) SSPs reported distribution of ≥10,000 naloxone doses during the last 12 months, accounting for 382,132 naloxone doses, 54% of all doses distributed by SSPs in the past 12 months. These 14 SSPs are located throughout six of the nine census divisions. Seventy-two (29%) SSPs ran out of naloxone or needed to ration their naloxone in the preceding 3 months.

The reach of SSP-based OEND programs varied by U.S. Census division. The highest ratios of persons who received naloxone per opioid overdose death (13:16) and numbers of naloxone doses distributed per opioid overdose death (22:37) were from SSP-based OEND programs in the Mountain, Pacific, and West North Central U.S. Census divisions; SSP-based OEND programs in the East South Central, Middle Atlantic, New England, and South Atlantic U.S. Census divisions had low ratios of persons provided naloxone per opioid overdose death (1:6) and of naloxone doses distributed per opioid overdose death (4:10). SSP-based OEND programs in the East North Central division achieved a high ratio of naloxone doses distributed per opioid overdose death (24), but a low ratio of persons provided naloxone (four) per opioid overdose death. The U.S. Census divisions with higher opioid overdose death rates included the East North Central, East South Central, Middle Atlantic, New England, and South Atlantic divisions (Figure 2).

**FIGURE 2:**
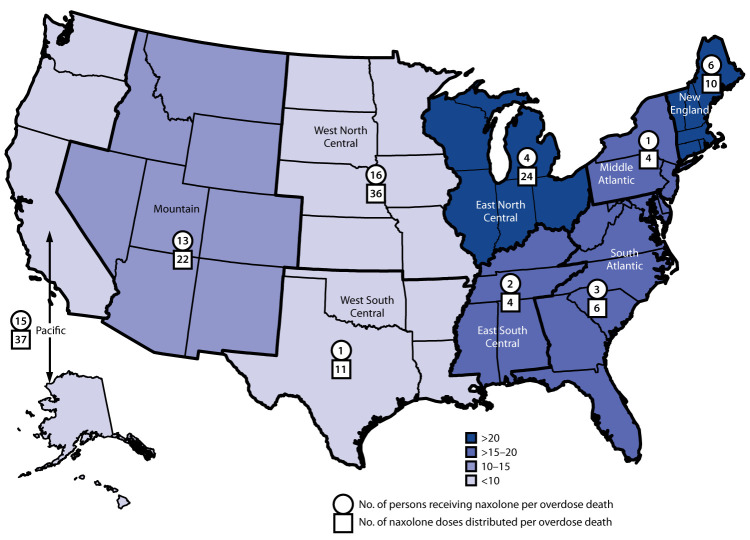
Reach of syringe service program (SSP)–based overdose education and naloxone distribution programs,*^,†^ by U.S. Census division (N = 247 SSPs), 2019 * SSPs were asked how many people received naloxone and how many naloxone doses were distributed in the past 12 months from their program. Opioid overdose deaths and opioid overdose death rates were from 2017 National Center for Health Statistics (https://www.cdc.gov/nchs/index.htm) data. Data were geocoded to the census division where the SSP was based, not necessarily where the naloxone was distributed nor residence of the persons provided naloxone. ^†^ Participating SSPs were identified by using the North America Syringe Exchange Network database.

## Discussion

As of 2019, 247 (94%) of 263 SSPs responding to an online survey had implemented OEND, marking a substantial increase from a 2013 survey that found that 55% of SSPs had implemented OEND (*5*). However, the bulk of naloxone distribution, in terms of the number of persons provided naloxone and the number of naloxone doses dispensed, were delivered by only 14 (6%) SSPs. Although SSPs are responding to different needs in the locations where they operate, this finding suggests the geographic distribution of SSP-based OEND delivery is highly concentrated in certain areas.

The existence of OEND within SSPs does not assure that the benefits of naloxone have been sufficiently and consistently extended in those areas, and there is currently no consensus regarding how many persons should receive naloxone or how many naloxone doses should be distributed, given the underlying need. A study from Massachusetts reported a 46% reduction in opioid overdose mortality when communities enrolled >100 persons at risk for experiencing or observing an overdose per 100,000 population into OEND programs (*7*). Research from Scotland demonstrated a 62% reduction in the opioid overdose mortality rate when the national program distributed 20 times the number of naloxone doses as the previous year’s number of opioid overdose deaths (*8*). Optimizing SSP-based OEND programming might require maximizing the number of participants provided naloxone and the number of naloxone doses distributed to participants. The reach of U.S. SSP-based OEND (as measured by the number of persons provided naloxone and the number of naloxone doses distributed per the number of opioid overdose deaths during the preceding year), was highest in the Mountain, West, and West North Central U.S. Census divisions. However, SSPs in the eastern part of the United States had high opioid overdose death rates but low ratios of persons provided naloxone or naloxone doses relative to the previous year’s opioid overdose deaths. Scaling up SSP-based OEND programming in these areas of the country is important; ensuring that SSPs have adequate resources and staffing, as well as supportive legal environments, might be a critical component to achieving these goals.

The findings in this report are subject to at least five limitations. First, other SSPs might exist that are not included in NASEN’s database. Second, the survey response rate was 77%; however, previous reports have shown that SSPs that do not participate tend to be small programs (*9*); therefore, it is likely that the larger programs are represented in this analysis. Third, although the online survey might have reduced response bias, responses were self-reported and not validated with programmatic records. Fourth, some organizations provided estimates for the number of naloxone doses distributed and the number of persons provided naloxone, which could result in under- or overreporting. Finally, SSPs operate on a smaller scale than U.S. Census divisions; therefore, the geographic distribution of naloxone distribution is not uniform within them. Further, SSP-based OEND delivery is concentrated where SSPs operate, especially those SSPs distributing ≥10,000 naloxone doses. In this analysis, the number of responding SSPs varied by U.S. Census division; however, the 14 SSPs that accounted for approximately one half of OEND distribution were located throughout six of the nine U.S. Census divisions.

This study found high levels (94%) of OEND implementation within SSPs in the United States; however, the number of persons provided naloxone and the number of naloxone doses distributed varied substantially across SSPs in U.S. Census divisions. Opportunity exists to improve the reach of SSP-based OEND programs, especially in areas of the country with high opioid overdose mortality rates. The introduction of fentanyl into the illicit drug supply has resulted in a sharp increase in the overdose rate in many regions, including those with longstanding SSP-based OEND programs (*10*). Ensuring that all SSP participants are provided access to a sufficient and consistent supply of naloxone over time can optimize efforts to reduce opioid overdose deaths. Public health initiatives might be enhanced with efforts to scale-up SSPs throughout the United States.

**Summary**

**What is already known about this topic?**

In 2013, 55% of U.S. syringe service programs (SSPs) had implemented overdose education and naloxone distribution (OEND).

**What is added by this report?**

In 2019, among 263 SSPs responding to an online survey, 247 (94%) had implemented OEND. The number of persons who received naloxone per opioid overdose death and the number of naloxone doses distributed per opioid overdose death during the previous year varied by census division.

**What are the implications for public health practice?**

Maximizing participants engaged in OEND and naloxone doses distributed to SSP participants might help to optimize SSP-based OEND programming. Scaling up SSP-based OEND delivery could be a critical component for areas of the country with high opioid overdose death rates.

## Footnotes

* https://www.cdc.gov/ssp/index.html.

† https://www2.census.gov/geo/pdfs/maps-data/maps/reference/us_regdiv.pdf.

§ https://www.nasen.org/.
